# Differential RNA Expression Profile of Skeletal Muscle Induced by Experimental Autoimmune Myasthenia Gravis in Rats

**DOI:** 10.3389/fphys.2016.00524

**Published:** 2016-11-10

**Authors:** Henry J. Kaminski, Keiichi Himuro, Jumana Alshaikh, Bendi Gong, Georgiana Cheng, Linda L. Kusner

**Affiliations:** ^1^Department of Neurology, George Washington UniversityWashington, DC, USA; ^2^Department of Neurology, Graduate School of Medicine, Chiba UniversityChiba, Japan; ^3^Department of Pediatrics, Washington UniversitySt. Louis, MO, USA; ^4^Department of Pathobiology, Cleveland ClinicCleveland, OH, USA; ^5^Pharmacology and Physiology, George Washington UniversityWashington, DC, USA

**Keywords:** myasthenia gravis, acetylcholine receptor, autoimmunity, gene expression profiles, skeletal muscle, metabolism

## Abstract

The differential susceptibility of skeletal muscle by myasthenia gravis (MG) is not well understood. We utilized RNA expression profiling of extraocular muscle (EOM), diaphragm (DIA), and extensor digitorum (EDL) of rats with experimental autoimmune MG (EAMG) to evaluate the hypothesis that muscles respond differentially to injury produced by EAMG. EAMG was induced in female Lewis rats by immunization with acetylcholine receptor purified from the electric organ of the *Torpedo*. Six weeks later after rats had developed weakness and serum antibodies directed against the AChR, animals underwent euthanasia and RNA profiling performed on DIA, EDL, and EOM. Profiling results were validated by qPCR. Across the three muscles between the experiment and control groups, 359 probes (1.16%) with greater than 2-fold changes in expression in 7 of 9 series pairwise comparisons from 31,090 probes were identified with approximately two-thirds being increased. The three muscles shared 16 genes with increased expression and 6 reduced expression. Functional annotation demonstrated that these common expression changes fell predominantly into categories of metabolism, stress response, and signaling. Evaluation of specific gene function indicated that EAMG led to a change to oxidative metabolism. Genes related to muscle regeneration and suppression of immune response were activated. Evidence of a differential immune response among muscles was not evident. Each muscle had a distinct RNA profile but with commonality in gene categories expressed that are focused on muscle repair, moderation of inflammation, and oxidative metabolism.

## Introduction

Myasthenia gravis (MG) is caused by antibodies, primarily directed at skeletal muscle nicotinic acetylcholine receptor (AChR), which lead to a reduction of AChR number and damage of the muscle endplate, producing a failure of neuromuscular transmission that results in weakness (Engel et al., 1976). The pathophysiology would be expected to compromise muscles to a similar extent, but clinical investigation for over a 100 years have demonstrated a preferential involvement of certain muscles. Explanations for the differential targeting may lie in subtle aspects of the antibody-antigen engagement *in vivo* but are more likely to entail variations in the properties of the targeted muscles.

The differential involvement of skeletal muscles by neuromuscular disorders, including MG, is poorly understood but likely is a function of disease specific pathophysiology and properties of the individual muscles. In particular, differences in functional requirements of a muscle impact the gene expression pattern. In its role in eye movement extraocular muscle (EOM) is constantly, and this is reflected in its transcriptional profile differing from jaw and leg muscle in expression of glycogenic and gluconeogenic genes (Porter et al., 2001; Fischer et al., 2005). Further, lactate is a significant substrate for EOM, which is in stark contrast to other skeletal muscles that excess lactate produces fatigue (Andrade and McMullen, 2006). Similarly, as reflected in fiber-type distribution diaphragm also possess properties that support its high energy requirements compared to leg muscles (Polla et al., 2004). The consequences of neuromuscular disorders on whole body metabolism may then also be expected to differentially impact muscles.

Extraocular muscle (EOM) are preferentially involved by MG and several non-exclusive explanations have been proposed. A patient may develop dramatic double vision with even minimal weakness of an EOM, but a similar level of weakness of another muscle would not produce clinically evident symptoms. The extremely rapid firing rate of ocular motor neurons and the immature appearance of their neuromuscular junctions may place the EOM at particular risk for a neuromuscular transmission disorder. The RNA expression profiles of EOM, extensor digitorum longus (EDL), and diaphragm (DIA) muscle from rats with passively-transferred MG (PTMG) (Kusner et al., 2015) produced by administration of acetylcholine receptor antibody supports a greater degree of injury to EOM (Zhou et al., 2014), which supports that EOM has unique immunological characteristics that places them at specific risk for MG (Kaminski et al., 2004; Soltys et al., 2008; Pedrosa Domellof, 2013).

EAMG induced in rodents by immunization with purified AChR mimics the human disease much better than administration of AChR antibodies (Losen et al., 2015). Within 6 weeks of a single immunization, rats generate AChR antibodies and then weakness, which improves with cholinesterase inhibition. As with humans, infiltrates of inflammatory cells are not prominently observed in muscle (Nakano and Engel, 1993; Baggi et al., 2012), which is in contrast to PTMG. In order to assess, variations in intrinsic response of muscles to EAMG, we used RNA expression profiling of diaphragm (DIA), extensor digitorum longus (EDL), and EOM to assess their response.

## Materials and methods

### Ethics statement for animal use

Six to eight week old female Lewis rats weighing 120–150 g (Harlan, Indianapolis, IN) were maintained in the Case Western Reserve University School of Medicine animal facility. The animal facility follows IACUC, AAALAT, and AALAS standards concerning appropriate housing, cage cleaning procedure, air purity, feed, temperature, humidity, light and dark cycle. Animals were housed in isolator cages in a pathogen-free environment, and rodent chow and water were provided *ad libitum*. A veterinarian is on staff and will be observing the health of the animals throughout the study. All animal studies were conducted according to protocol approved by the Case Western Reserve Institutional Animal Care and Use Committee Approval Number 030185. All efforts were made to minimize animal suffering. Tissue was harvested after euthanasia by CO_2_ asphyxiation.

### Induction and evaluation of EAMG

*Torpedo* AChR was purified from the electric organ of *Torpedo californica* by affinity chromatography as previously described (Lindstrom et al., 1983). Rats were immunized once at the base of the tail by subcutaneous injection of purified *Torpedo* AChR (40 μg/rat in 200 μl) emulsified in complete Freund's adjuvant supplemented with additional non-viable *Mycobacterium tuberculosis* H37RA (0.5 mg/rat; Difco Laboratories, Detroit, MI). Control rats were immunized with the same volume of adjuvant without AChR. Rats were monitored for evidence of weakness and their status scored based on a commonly used motor strength scale, as follows: 0 = can grip and lift lid of a cage, 1 = can grip but cannot lift the lid of a cage, 2 = unable to grip cage lid, 3 = unable to grip and has hind limb paralysis, 4 = moribund. Weight was assessed initially on a bi-weekly basis and then every other day when weakness or weight loss became evident.

### Tissue preparation

After euthanasia, EOM rectus muscles, DIA, and EDL muscles were dissected from rats 6 weeks after initiation of the experiment. Muscles were pooled from 4 to 5 rats for each of three independent replicate groups. The study was then repeated twice to produce the 3 replicates for the array analysis. This procedure served to limit inter-animal and inter-experiment variability. Tissues were snap frozen in liquid nitrogen and stored at −80°C until use.

### Serum AChR antibody determination

Blood was obtained at week 2 by tail vein puncture and after euthanasia from the heart at week 6. Serum was isolated and AChR antibody determination made by ELISA. Ninety-six-well immune-plates (Corning; New York, NY) were saturated with 200 μl (10 μg/ml AChR) in PBS buffer (0.1% Tween20 in PBS) per well and incubated overnight at 4°C. After washing twice with PBST buffer, the plates were incubated with 200 μl of blocking buffer (5% of bovine serum albumin in PBS) per well at 37°C for 0.5–1.5 h. The plates were washed twice with PBST buffer and then incubated for 1 h at 37°C with 100 μl of the diluted test serum (1:200). The plates were then washed twice with PBST, each well received 100 μl of peroxidase-labeled rabbit anti-rat IgG and incubated for 1 h at 37°C. One hundred microliter of substrate solution [0.05 M citrate, 0.1 M NaCl/Pi, 2,20-Azino-bis(3-ethylbenzothiazoline-6-sulfonic acid), 0.03% H_2_O_2_] was incubated for 15 min at 37°C. Color development was measured at 405 nm using a microplate reader.

### Immunohistochemistry

For analysis of C9 deposition at neuromuscular junctions, cryosections of muscles were prepared. Sections were incubated with rabbit anti-ratC9 (gift of M. E. Medof) and then double-stained with FITC-labeled goat anti-rabbit Ab and Texas red–labeled αBTX (2 μg/ml; Molecular Probes Inc., Eugene, Oregon, USA) to identify neuromuscular junctions. Sections were examined with a Nikon Diaphot fluorescence microscope (Nikon Instruments Inc., Melville, New York, USA) and analyzed using ImagePro software (Media Cybernetics, Silver Spring, Maryland, USA).

### Sample preparation for microarrays

The muscle harvested from DIA, EDL, and EOM of four rats pooled from four EAMG or control rats during RNA isolation forming three samples for subsequent array analysis. Total RNA was extracted using TRIzol reagent (GibcoBRL, Rockville, MD). RNA pellets were cleaned by RNasey kits and re-suspended at 1 mg RNA/ml DEPC-treated water and 5 μg was used in a reverse transcription reaction (SuperScript II; Life Technologies, Rockville, MD) to generate first strand cDNA. Double strand cDNA was synthesized and used in an *in vitro* transcription (IVT) reaction to generate biotinylated cRNA. Fragmented cRNA (15 μg) was used in a 300 μl hybridization cocktail containing herring sperm DNA and BSA as carrier molecules, spiked IVT controls, and buffering agents. A 200 μl aliquot of this cocktail was used for hybridization to Affymetrix rat REA230 (Santa Clara, CA) microarrays for 16 h at 45°C. The manufacturer's standard post-hybridization wash, double-stain, and scanning protocols used an Affymetrix GeneChip Fluidics Station 400 and a Hewlett Packard Gene Array scanner.

### Microarray data analysis

Raw data from microarray scans were analyzed with Affymetrix GCOS 2.0. GCOS evaluates sets of perfect match (PM) and mismatch (MM) probe sequences to obtain both hybridization signal values and present/absent calls for each transcript. Microarrays were scaled to the same target intensity and pairwise comparisons were made between experimental and control samples. Transcripts defined as differentially regulated met the criteria of: (a) consistent increase/decrease call across 7 out of 9 replicate comparisons, based upon Wilcoxon's signed rank test (algorithm assesses probe pair saturation, calculates a *p*-value and determines increase, decrease, or no change calls). Any transcripts with expression intensity below 400 (5 time of background level) across all the samples were also excluded since distortion of fold difference values results when expression levels are low and may be within the level of background noise. Data were visualized as a hierarchical cluster analysis generated (Genespring software, version 7.2; Silicon Genetics, Redwood city, CA). Annotation was done according to Affymetrix NetAffyx Gene Ontology database. Data for the 18 microarray experiments used in this report can be found in the National Center for Biotechnology Information (NCBI) Gene Expression Omnibus (GEO), series accession number GSE11465.

### Quantitative real-time PCR (qPCR)

Select transcripts were reanalyzed by qPCR, using the same samples as in the microarray studies. Transcript-specific primers (Supplemental Table 1) were designed using Primer Express 2.0 software [Applied Biosystems, Inc. (ABI), Foster City, CA] and specificity confirmed by NCBI BLAST. Reverse transcription was carried out on 1 μg total RNA with *In vitro* Reverse transcription reagent. qRT-PCR used SYBR green PCR core reagent in 24 μl volume, with an ABI PRISM 7000 Sequence Detection System. GAPDH was used as an internal positive loading control. Fold change values represent averages from triplicate measurements, using the 2^−ΔΔCT^ method (Simon, 2003).

## Results

### Confirmation of EAMG induction

After the immunization, serum AChR antibody levels of the experimental group were 2.5 μg/ml ± 1.1 (2 weeks) and 8.05 μg/ml ± 3.38 (6 weeks). AChR antibody was undetectable in the control group throughout the experiment, while all rats in the experimental group developed elevations of AChR antibody levels. At weeks 4–5, weight loss was observed in EAMG rats and weakness became evident as assessed by reductions of grip strength and reduction of observed movement (data not shown). At week 6 experimental rats had a mean weight loss of 11.5% ± 3.45 compared to their peak weight, while control rats had a mean weight gain of 5.9% ±1.0 compared to the previous week weight. All rats reached the end of the experiment with no need for early euthanasia. To assess for activation of the complement system, we evaluated C9 deposition from control and experimental rats. Endplates from all control muscles had α-BTX staining and no C9 staining. Endplates from all EAMG muscles demonstrated endplates C9 deposition which overlapped with α-BTX fluorescence (not shown).

### RNA profile analysis

To identify global alterations in gene expression related to EAMG in EOM, DIA, and EDL, RNA was prepared and processed for microarray hybridization from AChR immunized and control CFA immunized rats. The percentage of transcripts detected as present in each sample ranged from 48 to 63.7 with average 53.3 ± 4.2. The GAPDH probes 3–5 ratio ranged from 1.06 to 2.66 with average 1.27 ± 0.35, indicating RNA quality was appropriate and the hybridization was successful among the samples. The results are consistent with our previous expression profiling studies (Zhou et al., 2014).

**Figure 1:**
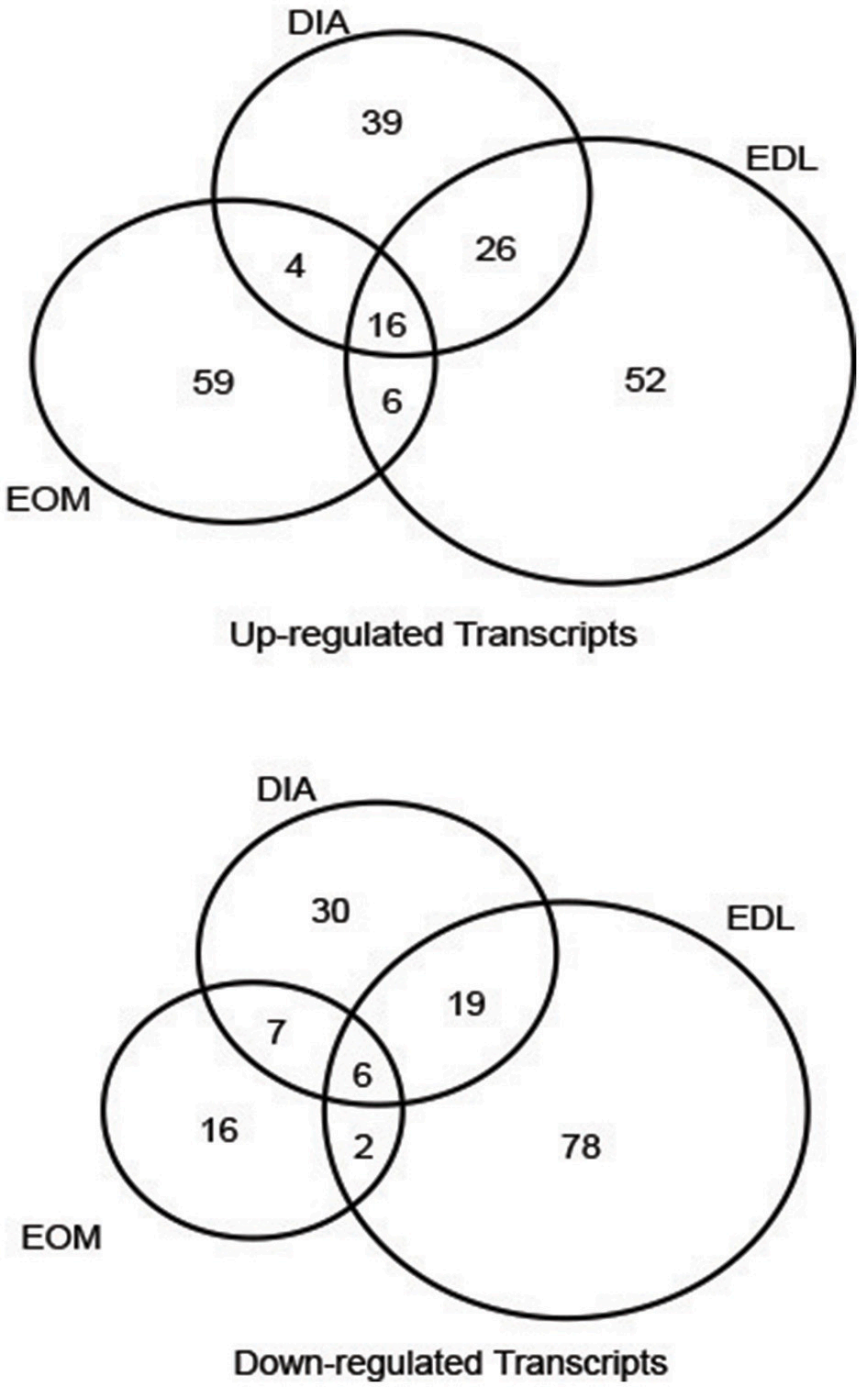
**Venn diagrams showing the numbers of differentially expressed transcripts in EAMG muscles compared with control rats shared by or unique to DIA, EDL, and EOM**.

**Figure 2:**
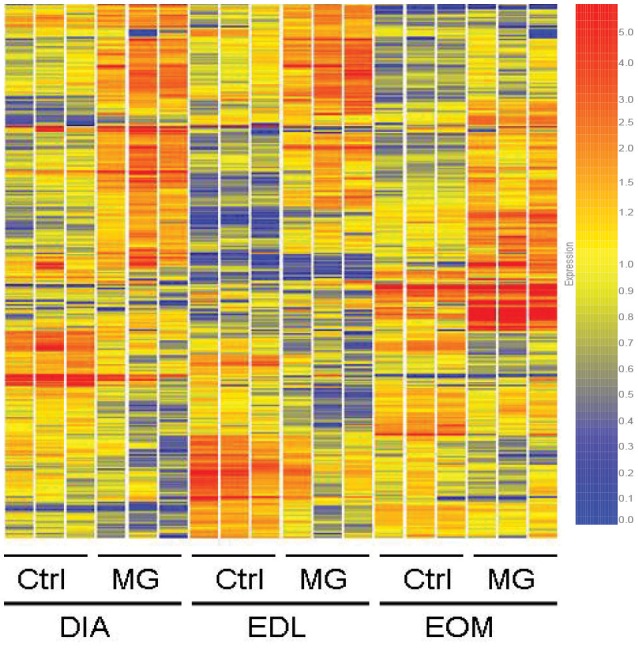
**Hierarchical cluster analysis using 359 gene probes identified as differentially expressed in EAMG among DIA, EDL, and EOM**. The three independent replicates of each group are represented. Expression ratios are color-coded. The scale at the right denotes normalized expression levels (*red*, high expression; *blue*, low expression).

We found a total of 359 transcripts (Figure 1) altered by 2-fold in 7 of 9 series pairwise comparisons between the experimental and control groups among the muscles. Figure 1 shows the degree of overlap in gene differences across the three muscles. DIA showed 147 gene changes, 85 genes were up-regulated and 62 down-regulated, EDL had 205 genes changed, 100 were up-regulated and 105 down-regulated, and EOM have 116 changed, 85 are up-regulated and 31 down-regulated. Using the 359 transcripts, hierarchical cluster analysis (Figure 2) demonstrated a distinct expression pattern of the genes found to be differentially influenced by EAMG across the three muscles. Comparison of the differentially expressed transcripts identified 16 upregulated and 6 downregulated transcripts shared among the three muscles (Table 1). Functional annotation of the differentially expressed genes identified more than a half are involved in signal transduction, metabolism and transcription regulation, suggesting the muscles experience adaptation to EAMG directly and to metabolic alterations associated with the disease (Table 2, Supplemental Table 2). Alteration of expression of 10 genes (*Enc1, Nfkbia, Nfix, Errfi1, Glipr2, Dyrk2, Hbb, Hba-a1, Pnpla2, Galnt12*) were identified common to the three muscles as a response to EAMG. The roles of these genes fell into two broad categories: muscle repair and suppression of inflammatory signals. The Discussion provides detailed consideration of expression alterations.

**Table 1:** **Gene transcript changes common to all muscles**.

**Symbol**	**Name**	**DIA**	**EDL**	**EOM**	**Classification**
Ankrd1	Ankyrin repeat domain 1	5.6	5.5	3.6	Transcription
Cebpd	CEBP delta	2.9	8.4	2.5	Transcription
Nfix	Nuclear factor I/X	−4.7	−2.0	−2.9	Transcription
Nfkbia	Nuclear factor of kappa light chain	2.3	2.8	2.3	Transcription
Ddit4	DNA-damage-inducible transcript 4	3.1	4.7	3.1	Stress
Gadd45a	Growth arrest and DNA-damage-inducible 45 alpha	2.3	2.1	2.5	Stress
Mt1a	Metallothionein 1a	4.7	8.2	3.3	Stress
Errfi1	ERBB receptor feedback inhibitor 1	2.9	3.0	2.8	Stress
Mt1e	Metallothionein 1e	3.0	5.5	2.8	Stress
Gpnmb	Glycoprotein (transmembrane) nmb	2.2	3.1	5.2	Signal
Glipr2	Golgi- associated PR-1 protein	−2.4	−2.4	−2.2	Signal
Dyrk2	Dual specificity tyrosine-phosphorylation-regulated kinase 2	−2.4	−2.3	−2.4	Signal
Enc1	Ectodermal-neural cortex 1	2.4	2.1	2.1	Signal
Hbb	Hemoglobin beta chain complex	−4.8	−2.4	−3.9	Metabolism
Neu2	Neuraminidase 2	−2.9	−4.0	−2.2	Metabolism
Hba-a1	Hemoglobin alpha 2 chain	−3.6	−2.3	−3.5	Metabolism
Pnpla2	Patatin-like phospholipase domain containing 2	2.4	2.8	2.1	Metabolism
pdk4	Pyruvate dehydrogenase	2.4	3.5	2.3	Metabolism
fmo2	Flavin containing monooxygenase 2	4.0	3.0	3.9	Metabolism
galnt12	GalNAc transferase 12	3.5	2.8	2.6	Metabolism
Angptl4	Angiopoietin-like 4	6.4	6.2	17.5	Metabolism
Fkbp5	FK506 binding protein 5	4.5	5.3	5.6	Immune

**Table 2:** **Transcript changes in percent by category**.

	**Shared**	**EOM**	**DIA**	**EDL**
Transcription	18	5	7	19
Stress	22	0	1	5
Metabolism	32	21	6	17
Signaling	18	33	44	25
Proteolysis	0	5	4	5
Muscle-related	0	4	1	5
Inflammatory	5	8	7	2
^*^ECM	5	3	0	2
EST/Unknown	0	21	30	20

* *Extracellular Matrix*.

**Figure 3:**
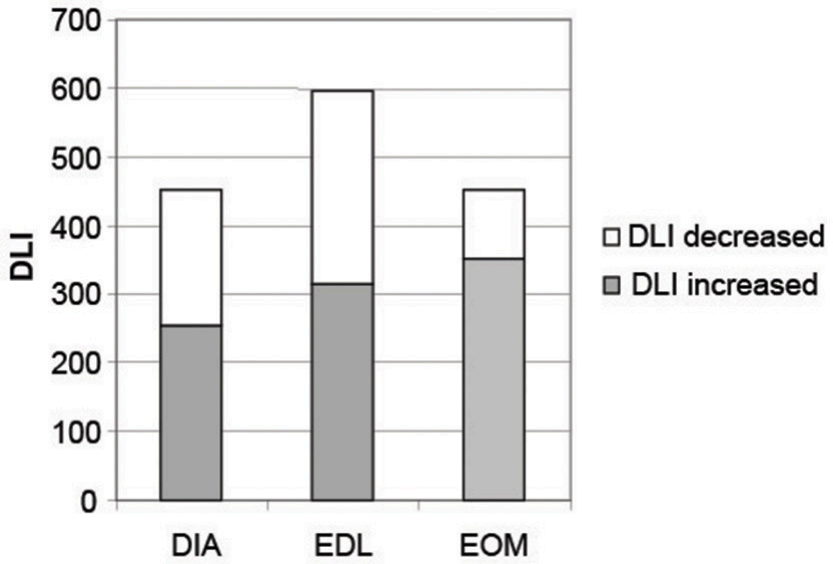
**The aggregate DLI of the three muscles for EAMG illustrate disease progression by summing the absolute values of fold changes of differentially regulated transcripts in each muscle**.

In our previous study of passive transfer MG and a study of mdx mice (Porter et al., 2003a; Zhou et al., 2014), we determined a disease load index (DLI), which sums the absolute fold change values of increased and decreased transcript to provide a single transcriptional index of EAMG pathology. In the present investigation, EDL had the greatest DLI (Figure 3), although EOM had the greatest total of increased transcript levels with 351 total fold increase to 101 total fold decrease. The greater DLI of EDL suggests a greater transcriptional response to EAMG than EOM or DIA.

### Validation of RNA profile

We used qPCR to validate results of the RNA profiling. We determined expression levels of 14 transcripts to provide a broad assessment of the array results. Three upregulated transcripts (*Ankrd1, Mt1a, Cebpd1*) and one down regulated transcript (*Neu2*) that by RNA profiling had been identified as altered across all three muscles. We assessed transcripts that were previously identified as altered by EAMG (*Chrna1, Pde4b, Cts1, Trm63*) (Mizrachi et al., 2010; Zhou et al., 2014) or involved in inflammation (*NF*κ*B, Ctse*). We evaluated transcripts that were increased in the RNA profiling of EOM alone (*Plunc, Pax6, Rgs2*) (Porter et al., 2003b) or known to be increased in EOM (*Csrp3*) (Diehl et al., 2006). RNA profiling and qPCR results correlated well with correlation coefficient of 0.9 (Table 3).

**Table 3:** **Fold change comparison between real-time PCR and microarray**.

**Gene symbol**	**DIA**		**EDL**		**EOM**	
	**qPCR**	**Array**	**qPCR**	**Array**	**qPCR**	**Array**
*ANKRD1*	11.7	6.2	8.1	5.5	5.6	3.2
*MT1A*	4.2	4.7	4.8	8.2	5.3	3.4
*CEBPD1*	5.3	3.0	6.5	4.5	3.3	2.2
*PDE4B*	3.2	2.0	2.8	1.7	2.0	1.3
*CTS L*	2.8	1.8	2.7	2.8	2.2	1.4
*CHRNA1*	2.1	1.9	2.5	1.7	3.7	2.3
*CTS E*	1.9	1.2	0.9	1.0	7.5	7.6
*CSRP3*	5.9	2.4	5.4	3.3	3.0	1.4
*NEU2*	−3.2	−2.7	−3.8	−4.0	−1.9	−2.2
*NFκB*	1.1	1.0	1.8	1.3	1.3	1.0
*PAX6*	N/A	0.5	N/A	1.3	6.7	4.0
*PLUNC*	N/A	0.9	N/A	1.0	4.1	3.0
*RGS2*	1.4	1.1	1.0	0.8	−1.8	−1.3
*TRIM63*	6.3	2.4	4.3	2.5	1.3	1.0

## Discussion

We found distinct genomic signatures for DIA, EDL, and EOM in response to EAMG with a small number of gene alterations shared among the muscles (Figure 1). The results of array analysis were validated by qPCR with a significant correlation. Across the muscles, there was wide variation in the specific gene changes, but a commonality of genes that were altered occurred in cell signaling, transcriptional factors, and metabolism categories. Genes associated with muscle injury repair were upregulated. Only, a few genes primarily associated with the immune system or inflammation were modified by EAMG, however these were focused on genes that would suppress inflammation. The observation is consistent with histology of muscle of human MG, and EAMG in the chronic stage, which does not have evidence of inflammation (Nakano and Engel, 1993; Losen et al., 2015; Tuzun et al., 2015). The expression of immunosuppressive genes provides the first insight into why EAMG, by extension human MG, lacks inflammatory infiltrates in muscle after the acute induction stage.

A critical issue that cannot be addressed by our investigation is the degree to which individual mechanisms alter transcriptional profiles. For example, reduced muscle activity produced by EAMG, or for that matter, passive transfer MG in our previous investigation influences the gene expression pattern. The EOM in particular have unique neuromuscular transmission properties with synapses subject to extremely high stimulation rates by their motor neurons and a reduction in muscle stimulus would likely lead to transcriptional alterations (Spencer and Porter, 2005). Knowing that post-synaptic damage is likely to influence presynaptic properties, we cannot dissociate our results to one specific alteration produced by EAMG (Ouanounou et al., 2016). As we discuss the influence of alterations in whole body metabolism produced by weight loss further will alter gene expression of each muscle in a differential pattern given their unique characteristics.

### Gene expression alterations unique to EAMG and common to all muscles

The enhanced expression of five genes (*Nfix, Enc, Errfi1, Glipr2, Dyrk2, Galnt12*) modified by EAMG appear to enhance muscle repair. *Nfix*, is a transcription factor known to be involved in myogenesis and muscle regeneration (Déak et al., 2014). *Enc1* is an actin-binding protein, which is involved in neuronal process formation (García-Calero and Puelles, 2005; Kim et al., 2009). Its expression has not been observed previously in muscle, and presumably, *Enc1* would function to aid recovery of damaged neuromuscular junctions. *Errfi1* (also known as *Mig6*) is an inhibitor of epidermal growth factor signaling and is increased with cell stress and positively regulates cell growth. Elevation of *Errfi1* expression would be expected as a response to muscle injury, either from increased oxidative metabolism or neuromuscular junction injury (Pante et al., 2005). Our previous PTMG profiling study identified a homolog of *Mig6* to be upregulated across all three muscles. *Glipr2* is a signaling molecule, which has not been well-characterized, but is upregulated in sciatic nerve injury from experimental diabetes (Zhang et al., 2015). *Glipr2* is involved in transition of epithelial to mesenchymal cells by way of epidermal growth factor signaling pathways (Huang et al., 2013), which are also known to be involved in recovery from muscle injury. *Dyrk2* encodes a tyrosine kinase, which negatively regulates growth of cardiac myocytes (Weiss et al., 2013), and the reduced expression of *Dyrk2* in EAMG would be expected to promote recovery from injury. *Galnt12* encodes an acetylgalactosaminyl transferase and polymorphisms in the gene are associated colonic cancer (Clarke et al., 2012; Wang et al., 2014). The increased expression of *Galnt12* likely enhances protein modifications that may be involved in the regenerative process.

*Nfkbia* (nuclear factor of kappa light polypeptide gene enhancer in B-cells inhibitor, alpha) is a transcriptional factor with increased expression among all three muscles exclusive to EAMG. *Nfkbia* inhibits cell apoptosis by inhibition of caspase. In rats, *Nfkbia* is decreased in diet-induced obesity and hyperlipidemia. *Nfkbia* can inhibit NFκB by direct binding and regulation of transcriptional responses to NFκB, including cell adhesion, immune and proinflammatory responses, apoptosis, differentiation and growth (Hotamisligil, 2006). In addition to its effects on contractility, *Fkbp5* modulates NFκB activity and in tandem with *Nfkbia* would reduce inflammatory signaling in muscle (Erlejman et al., 2014).

In addition, to the primary categories of muscle repair and immunosuppression, there were three gene changes that would influence metabolism. The *Dyrk2* gene product phosphorylates glycogen synthase (Skurat and Dietrich, 2004) and therefore, the gene's down-regulation would be a response to a shift to fatty acid oxidation (see discussion below). *Pnpla2* encodes a lipase that hydrolyzes fatty acids from triacylglycerol and mutations of the gene produce a myopathy. The upregulation of the gene's expression is also in keeping with the overall shift to fatty acid oxidation (Henriksson, 1995). This change to oxidation is a reflection of decreased availability of glucose and would further be reflected in a reduction of muscle force generation. The reason for the reduced expression of the two hemoglobin gene transcripts (*Hbb, Hba-a1)* in muscle is not clear.

### Gene expression changes specific to EAMG

Consistent with overall profile, alterations of genes involved in metabolism were the most common category for EOM. The gene encoding the alpha subunit of the acetylcholine receptor was increased in response to EAMG in skeletal muscle an EOM previously (Asher et al., 1990; Léger et al., 2006). We evaluated expression of four genes by qPCR expressed at high levels in EOM, three of which appear to be responsive to injury. *Pax6* is a transcriptional regulator involved in eye development and during development influences muscle formation (Davis-Silberman et al., 2005). Its increased expression in EOM suggests that it may be responding to injury, perhaps through the elevated activity of muscle satellite cells in EOM (McLoon et al., 2004). *Rgs2*, a regulator of G-protein signaling, also appears to be involved quiescent stem cell renewal of muscle (Subramaniam et al., 2014). *Cspr1*, a member of the LIM protein family, has many functions in skeletal and cardiac muscle including myocyte differentiation (Vafiadaki et al., 2015). *Plunc* is expressed in nasal epithelial tissue and has a bactericidal effects suggesting a role in innate immunity (Liu et al., 2016). Its upregulation in EOM in response to EAMG suggests a response to antibody-mediated injury but otherwise is unclear.

### Gene expression alterations common to all muscles and PTMG

PTMG is produced by administration of either mono- or poly-clonal antibody specifically directed toward the autoantigen, in the present study the skeletal muscle AChR (Kusner et al., 2015). The model mimics the final effector pathway of autoantibody destruction observed in humans with MG. The onset of muscle injury is rapid and is accompanied by muscle inflammation not seen in the active model or the human disease. In our previous study of PTMG of EDL, DIA, and EOM (Zhou et al., 2014), RNA profiling demonstrated a greater number of gene alterations, a preponderance of immune-related gene expression alterations, and EOM had the greatest DLI. There were specific gene alterations common among the three muscles and interestingly among these, several were shared with our present RNA profile of EAMG (Zhou et al., 2014). These ten genes were *Ankrd1, Gpnmb, Cebpd, Pdk4, Angptl4, Ddit4, Gadd45a, Mt1a, Fmo2*, and *Fkbp5*.

#### Evidence of immunosuppressive response across muscles

*Ankrd1* has been found to be increased in response in several forms of muscle injury, including denervation, motor neuron disease, stretch, and starvation (Miller et al., 2003; Wu et al., 2011; Calvo et al., 2012). *Ankrd1* also down regulates NFκB as an anti-inflammatory signal (Liu et al., 2015), which would contribute to the lack of local muscle inflammation in EAMG, while its increase in PTMG may be a response to acute inflammation observed. Osteoactivin (*Gpnmb*) is a type I transmembrane glycoprotein that is expressed in numerous tissues including skeletal muscle. Osteoactivin has immunosuppressive effects (Schwarzbich et al., 2012) and promotes maintenance of innervation (Furochi et al., 2007), these properties would aid recovery from injury by EAMG. In contrast to the PTMG study in which an inflammatory infiltrate was present, we can be confident that the source of *Gpnmb* transcripts is from the skeletal muscle. The increase of *Cebpd*, a transcriptional factor known to reduce pro-inflammatory cytokines (Pedersen and Febbraio, 2008; Scheler et al., 2013), would also serve to limit muscle inflammation in response to EAMG (Moore et al., 2012). Increased expression of cortisone-regulated target genes also would indicate a general anti-inflammatory state, which would also be reflected in metabolic alterations.

### Metabolic alterations influenced by EAMG

The other genes in common across MG models and the three muscles under study were primarily involved in metabolism. At the time of euthanasia, rats were demonstrating weight loss, however, in comparison an RNA profile of rat gastrocnemius after fasting, the gene alterations observed with EAMG are distinct (de Lange et al., 2004). The pyruvate dehydrogenase kinase gene is a key regulatory enzyme in skeletal muscle involved in switching the primary energy source from glucose to fatty acids in response to physiological conditions. Transcription of the *PDK4* gene is activated by fasting in a tissue-specific manner (Wende et al., 2005; Yumuk, 2006) and after both short-term high-intensity and prolonged low-intensity exercise. *Angptl4* (an inhibitor of lipoprotein lipase) expression was increased, and its protein product is directly involved in reduction of glucose utilization, enhanced lipid metabolism, and increased insulin sensitivity during fasting (Dutton and Trayhurn, 2008; Staiger et al., 2009). Metallothioneins are low molecular weight, cysteine–rich zinc binding proteins and serve to protect cells after oxidative stress injury (Reinecke et al., 2006). *Mt1a* is a metallothionein, which is induced in skeletal muscle of animals in catabolic states and physiological stress situations (Lecker et al., 2004), which also result in elevated levels of glucocorticoids and reactive oxygen species. Another metallothionein, *Mt1e*, was elevated among all muscles in the present study, but not the PTMG investigation. The increase in flavin-containing mono-oxygenase 2 (*Fmo2*) also would serve as a response to an increase in reactive oxygen species in the switch to fatty acid oxidation (Krueger and Williams, 2005). These observations suggest that EAMG directly or indirectly may increase intracellular reactive oxygen species with muscle responding with a potential adaptive response to systemic metabolic alterations. The uniform response to stress is further confirmed by increased expression of *Ddit4* and *Gadd45a*. *Ddit4* is a DNA-damage-inducible transcript, inhibits mTOR (downstream kinase of IGF pathway) functional control of cell growth in response to energy stress (Sofer et al., 2005; Wang, 2006). *Gadd45a* is activated by physiological stress and DNA damage serving to modulate cell cycle arrest and apoptosis (Prosperi, 1997). Increased *Cebpd* expression occurs with denervation and food deprivation (Allen et al., 2010; Zhang et al., 2013). In skeletal muscle *Cebpd* expression would moderate myostatin expression and potentially produce muscle atrophy (Yang et al., 2005).

### Muscle contractility gene influences

The increase of *Fkbp5* (FK506 binding protein) may serve to moderate calcium activation through the ryanodine receptor (Krueger and Williams, 2005). In PTMG, muscle contractility is compromised to a greater extent than expected from the neuromuscular transmission defect alone. An increase in *Fkbp5* would reduce influx of calcium and negatively impact muscle contractility. Muscle force generation is also reduced in isolated muscle preparations treated with sera from patients with MG (Imai et al., 2011, 2012). In EDL, *Zfn28* was found to be increased. *Zfn28* is a RING zinc finger protein family member, which localizes to the Z-line and M-line lattices of myofibrils. *In vitro* binding studies indicate that *Zfn28* binds to titin near the region responsible for kinase activity. Since these family members can form heterodimers, this suggests that these proteins may serve as a link between titin kinase and microtubule-dependent signal pathways in muscle (Ng et al., 2003). Alterations in the signaling complex could also moderate contractility through alterations in elasticity.

*Neu2* was the only gene reduced in expression among all three muscles and common to the RNA profile of PTMG. *Neu2* encodes a glycohydrolytic enzyme that is primarily expressed in mature muscle (Miyagi and Yamaguchi, 2012). The function of *Neu2* has not been well characterized but is considered to enhance muscle regeneration and development, and its reduced expression in EAMG may compromise muscle repair.

### RNA profile across muscles

Numerous clinical observations demonstrate a differential effect of neuromuscular disorders on specific muscle groups. Our previous RNA expression analysis of PTMG showed that EOM had the greatest DLI, which we had considered consistent with the greater susceptibility of EOM to disease observed in human MG. In the PTMG, transcript alterations were largely related to immune activity. In EAMG, EDL had the greatest transcriptional response to EAMG but this was related to preponderance of metabolic alterations. EOM given their specialized function in moving the globe and small size would not contribute to whole body metabolic control in contrast to EDL and other large muscles, which are critical to regulation of glucose, fatty acid, and amino acid synthesis and utilization.

Regarding specific gene expression alterations of note, the AChR subunit α-subunit was increased consistent with previous EAMG investigations. The expression difference was likely detectable in EOM because of its high innervation ratio and the increased expression reserved to subsynaptic nuclei (Hippenmeyer et al., 2007). *Spp1* (osteopontin) was upregulated in EOM. OPN is a glycosylated phosphoprotein originally identified in bone matrix, but now found to be produced in many cell types. OPN is considered a pro-inflammatory cytokine, and increased levels have been associated with inflammatory muscle disease (Urganus et al., 2009; Niewold et al., 2010; Kim et al., 2012) and muscular dystrophy (Kyriakides et al., 2011). Its elevated expression is in distinction from the upregulation of an anti-inflammatory state of RNA profile, which would support the contention that EOM has a unique immune environment, which in certain conditions enhances susceptibility to autoimmune and inflammatory disorders (Soltys et al., 2008).

Metallothionein family members are highly induced by catabolic states where found to be elevated across all muscle groups (Sacheck et al., 2007). Foxo1a, a transcription factor, was increased in EDL and diaphragm by EAMG and is also increased in states of catabolism (Sacheck et al., 2007). Cathepsin L was elevated in EDL with EAMG which is also the case for muscle atrophy.

### Clinical relevance

As with any animal study there are limits in application of results to human disease. The rat and human immune systems possess unique characteristics, and the exogenous administration of autoantigen with adjuvant does not mimic the spontaneous development of the breakdown in tolerance of the human disease (Losen et al., 2015). However, the ultimate common pathway of antibody attack with complement activation is shared between EAMG and human MG. The subsequent influence of generalized weakness on the animal and human also are likely to have commonalities.

The EAMG rats developed moderate weight loss, which is likely driving the gene expression changes related to metabolism. The three muscles shared similar responses in shifting to from glycolytic to oxidative metabolism. In keeping with this alteration pathway the same response in terms of stress and free radical metabolism pathways, and a number of nuclear receptors target genes involved in lipid and glucose metabolism were altered by EAMG. Cortisone or dexamethasone target genes were induced and insulin-moderated pathways were inhibited. This was true for EOM, which relies on glucose and lactate for generation of energy (Porter et al., 2001; Andrade and McMullen, 2006). These observations demonstrate that RNA profiles related to metabolism undergo significant alterations by EAMG.

In muscle, there are four pathways for protein degradation: lysosomal proteases including the cathepsins; calcium-dependent proteases; cytosolic ATP-dependent (proteasome); and cytosolic ATP-independent proteolytic pathways. In EDL and DIA FBXO32(atrogin-1) and Trim63(MuRF-1) were upregulated, these two genes are E3 ubiquitin-ligase, which are involved in ubiquitin-proteasome proteolysis pathway and are markers of muscle atrophy (Clavel et al., 2006; Edström et al., 2006). Their common transcription factor, Foxo1a was also increased in expression (Léger et al., 2006; Nakashima et al., 2006). Proteasome pathway related genes were more prominently expressed in EDL compared with DIA and EOM. EDL can be considered a “standard” skeletal muscle, which participates in regulation of whole body metabolism. These data suggest the EAMG leads to accelerated proteolysis, which is also likely to occur among patients with significant weakness. Since the active model of EAMG more closely mimics MG in humans, it is likely that metabolic alterations should be a focus of investigation in clinical studies.

## Conclusion

Our study demonstrates the complex alterations occurring on a transcriptional level in response to the direct effects of acetylcholine receptor antibody attack on the neuromuscular junction as well as secondary influences on nerve-muscle communication and development of weight loss. In our investigation we cannot uncouple these effects. We show that alterations in metabolism related genes occurs, an anti-inflammatory response develops, and muscle repair programs develop. Despite an expectation that EOM would have a greater DLI, this was not the case indicating that the reasons for the greater susceptibility of EOM to MG are not reflected in the transcriptional profile.

## Author contributions

The experimental work was performed in the laboratory of HK. The study was designed and supervised by HK. LK, GC, and BG performed animal experiments. BG and GC performed bioinformatics analysis. KH and JA assisted in data analysis. All authors participated in writing the initial drafts of the manuscript. All authors read and approved the final manuscript.

## Funding

Supported by National Institutes of Health R24EY014837 (HK).

### Conflict of interest statement

The authors declare that the research was conducted in the absence of any commercial or financial relationships that could be construed as a potential conflict of interest.
