# Supplementary material for: Differential RNA Expression Profile of Skeletal Muscle Induced by Experimental Autoimmune Myasthenia Gravis in Rats
Source: Front Physiol. 2016 Nov 10;7:524. doi: 10.3389/fphys.2016.00524 (PMC5102901; doi:10.3389/fphys.2016.00524)
Supplement: Supplementary file 1 [file Table1.DOC]

Supplemental Table 1. Primers for Real-time PCR

| Gapdh | 5-TGGTGGACCTCATGGCCTAC-3  5-CAGCAACTGAGGGCCTCTCT-3 |
| --- | --- |
| Ankrd1 | 5-TTGGCGATCGTGGAGAAGTTA-3  5-GCGTTTCCTCCACGACATG-3 |
| Mt1a | 5-CAAAGGTGCCTCGGACAAGT-3  5-GTACGGCAAGACTCTGAGTTGGT-3 |
| Cebpd | 5-CATCGACTTCAGCGCCTACA-3  5-CCGCTTTGTGATTGCTGTTG-3 |
| PDE4b | 5-CATAATCGGCCCCTCACATG-3  5-GTGATACGCCACATCAGAATGG-3 |
| Ctsl | 5-GGAAAATGGAGGTCTGGACTCA-3  5-CGTCGCTACAGGCTTCATGA-3 |
| Chrna1 | 5-GTCACCCACTTTCCCTTCGA-3  5-GGCTGGTCACTTTCCGGATT-3 |
| Ctse | 5-CACACGCACCACCTCTACCAT-3  5-GGTTGTGACGTGAGGCATAGC-3 |
| Csrp3 | 5-GGCTGCCTCAGCACAGACA-3  5-ATACCGACTTTCCGCATCGT-3 |
| Neu2 | 5-CAGCAGGACGGATGAGCAT-3  5-GGGTCACCACTTCCTCAGGTT-3 |
| NFkb | 5-CGCATCCGATTTTTGATAACC-3  5-TGCACTTTGTCACACAGCAAGA-3 |
| Pax6 | 5-CACCGCCCTCACCAACAC-3  5-ATAACTCCGCCCATTCACTGA-3 |
| Plunc | 5-CAAGTGAATATGCCCGTGGTT-3  5-TGGATCCTCCCCTGATTGTC-3 |
| Rgs2 | 5-GTTCGTCCCTGTCAGGAAAGC-3  5-GGGAAAGGTGTACAGCCAGCTA-3 |
| Trim63 | 5-GGGAAGACAGAACAAGGCTTTG-3  5-ACACGCCCTCTTCTTGATCCT-3 |
