# Supplementary material for: Differential RNA Expression Profile of Skeletal Muscle Induced by Experimental Autoimmune Myasthenia Gravis in Rats
Source: Front Physiol. 2016 Nov 10;7:524. doi: 10.3389/fphys.2016.00524 (PMC5102901; doi:10.3389/fphys.2016.00524)
Supplement: Supplementary file 2 [file Table2.DOCX]

# **Supplemental Table 2**. Summary of Gene Transcript Alterations

# **Transcript Alterations Specific to Diaphragm**

| **Accession No or** **Affymetrix ID** | **Gene** | **Mean Fold Difference** |
| --- | --- | --- |
| *Transcription* | | |
| LOC686314 | Hypothetical protein LOC387640 *DLN-1* | -2.0 |
| NM_001034125 | Period homolog 1 (Drosophila) *Per1* | 1.9 |
| NM_001135007 | Siah binding protein 1; FBP interacting repressor; pyrimidine tract binding splicing factor; Ro ribonucleoprotein-binding protein 1 *Siahbp1* | 1.7 |
| NM_001109425 | Zinc finger protein 703 *Zfp703* | 1.6 |
| NM_031135 | Kruppel-like factor 10 *Klf10* | 2.0 |
| *Stress* | | |
| NM_033285.3 | Tumor protein p53 inducible nuclear protein 1 *SIP27* | 1.8 |
| *Signal* | | |
| BF398673 | Golgi associated, gamma adaptin ear containing, ARF binding protein 2 *Gga2* | -1.7 |
| NM_001106472 | Dickkopf homolog 4 (Xenopus laevis) (predicted) *Dkk4_predicted* | -1.8 |
| NM_178095 | ATP-binding cassette, sub-family A (ABC1), member 1 *Abca1* | 1.5 |
| NM_001107887 | CD163 antigen *CD163* | 1.7 |
| NM_001108181 | Acyl-Coenzyme A dehydrogenase family, member 11 (predicted) *Acad11_predicted* | 1.7 |
| XM_001056825 /// XM_002725221 | Golgi phosphoprotein 2 (predicted) *Golph2_predicted* | 1.7 |
| NM_022707 | Phospholamban *Pln* | 0.1 |
| NM_171992 | Cyclin D1 *Ccnd1* | -2.2 |
| NM_199106 | UDP-N-acetyl-alpha-D-galactosamine:polypeptide N-acetylgalactosaminyltransferase 13 *Galnt13* | -1.1 |
| NM_001109470 /// NM_001135088 /// NM_001135089 | Coatomer protein complex, subunit zeta 1 (predicted) *Copz1_predicted* | -0.8 |
| NM_178095 | ATP-binding cassette, sub-family A *Abca1* | 1.8 |
| NM_001107898 | Solute carrier organic anion transporter family, member 5A1 (predicted) *Slco5a1_predicted* | -1.9 |
| NM_001105812 | Solute carrier family 43, member 2 (predicted) *Slc43a2_predicted* | 1.5 |
| NM_001106811 /// XM_003750455 /// XM_003754395 | A disintegrin-like and metallopeptidase (reprolysin type) with thrombospondin type 1 motif, 8 (predicted) *Adamts8_predicted* | 2.7 |
| None matched | Integrin beta 8 (predicted) *Itgb8_predicted* | 1.1 |
| NM_001106142 | Similar to actin filament associated protein; actin filament-associated protein, 110 kDa (predicted) *RGD1311580_predicted* | -0.8 |
| XR_008690 /// XR_086281 | Similar to GTL2, imprinted maternally expressed untranslated (predicted) *RGD1566401_predicted* | 1.9 |
| BF400606 | Sequestosome 1 *Sqstm1* | 1.8 |
| NM_001106142 | Similar to actin filament associated protein; actin filament-associated protein, 110 kDa (predicted) *RGD1311580_predicted* | -1.6 |
| NM_001044295 | Similar to tumor protein D53 (predicted) *D53* | 2.3 |
| NM_145677 | Solute carrier family 25 (mitochondrial carrier, phosphate carrier), member 25 *Slc25a25* | 1.8 |
| NM_001009617 | Coatomer protein complex, subunit gamma 2 *copg2* | -2.1 |
| NM_022499 | Parvalbumin *Pvalb* | -1.6 |
| NM_022707 | Phospholamban *Pln* | -1.5 |
| NM_001270867 /// NM_053601 /// NM_181687 /// NR_073089 | Neuronatin *Nnat* | -0.8 |
| NM_023991 | Protein kinase, AMP-activated, alpha 2 catalytic subunit *Prkaa2* | -1.8 |
| NM_017352 /// NM_031628 | Nuclear receptor subfamily 4, group A, member 3 *Nr4a3* | 1.8 |
| NM_053544 | Secreted frizzled-related protein 4 *Sfrp4* | -2.1 |
| NM_053955 | Crystallin, mu *Crym* | -0.8 |
| NM_019186 | ADP-ribosylation factor-like 4A *Arl4a* | 1.8 |
| *Proteolysis* | | |
| NM_001033652 /// NM_022630 | Transmembrane protease, serine 11d *Tmprss11d* | -2.0 |
| NM_017031 | Phosphodiesterase 4B, *Pde4b* | 1.7 |
| NM_053582 | Lipocalin 7 *Lcn7* | 1.8 |
| *Muscle* | | |
| NM_012604 | Myosin, heavy polypeptide 3, skeletal muscle, embryonic *Myh3* | 2.4 |
| Metabolism | | |
| NM_130739 | Acyl-CoA synthetase long-chain family member 4 *Acsl4* | 1.7 |
| NM_001108688 /// XM_003750066 | Cytidine deaminase (predicted) *Cda_predicted* | -1.8 |
| NM_031007 | Adenylate cyclase 2 *Adcy2* | 1.8 |
| NM_031543 | Cytochrome P450, family 2, subfamily e, polypeptide 1 *Cyp2e1* | 2.6 |
| *Inflammatory* | | |
| NM_001017496 | Similar to Small inducible cytokine B13 precursor (CXCL13) (B lymphocyte chemoattractant) (CXC chemokine BLC) *LOC498335* | 0.2 |
| AI411693 | Similar to immunoglobulin heavy chain 6 (Igh-6) /// similar to Ig H-chain V-region precursor /// similar to single chain Fv antibody fragment scFv 7-10A /// similar to Ig heavy chain V region MC101 precursor *RGD1359202 /// LOC299458 /// LOC314509 /// LOC366747* | 0.7 |
| L22655 | Similar to IG KAPPA CHAIN V-V REGION K2 PRECURSOR /// similar to NGF-binding Ig light chain *LOC500180 /// LOC500183* | 5.2 |
| NM_017020 | Interleukin 6 receptor, alpha *Il6ra* | 2.2 |
| NM_001008843 /// NM_001033986 | RT1 class I, CE5 *RT1-CE5* | 2.0 |
| *Unclassified* | | |
| 1391816_at | Unknown, transcribed | 3.1 |
| 1394503_at | Unknown, transcribed | 1.0 |
| NM_001107799 | Similar to dJ881L22.2 (novel protein) (predicted) *RGD1307696_predicted* | -1.6 |
| 1394020_at | Unknown, transcribed | -1.5 |
| 1393352_at | Similar to hypothetical protein MGC38960 (predicted) *RGD1310552_predicted* | 1.6 |
| 1389206_at | Transcribed locus, unknown | -0.8 |
| NM_001009709 | Similar to 1110007F12Rik protein *MGC109491* | 1.8 |
| 1389206_at | Transcribed locus *unknown* | -1.7 |
| BF544149 | Transcribed locus, strongly similar to XP_574268.1 PREDICTED: similar to LRRGT00194 [Rattus norvegicus] *unknown* | -1.9 |
| 1384802_at | Transcribed locus *unknown* | -1.1 |
| NM_001100811 | CDNA clone IMAGE:7323916 *mosc1* | 2.0 |
| 1382495_x_at | Transcribed locus *unknown* | -1.8 |
| 1382494_at | Transcribed locus *unknown* | -3.5 |
| BE110205 | Transcribed locus *unknown* | -1.4 |
| AI535567 | Transcribed locus *unknown* | -1.9 |
| BE108135 | EST | -2.0 |
| NM_001100811 | mitochondrial amidoxime reducing component 1 (Marc1) | 2.1 |
| NM_001134604 | Cytokine like (Cytl4) | 1.8 |
| NM_001106838 | Similar to hypothetical protein MGC38960 (predicted) *RGD1310552_predicted* | 1.8 |
| NM_001127484 | WW domain binding protein 1-like (wbp1l) | 1.9 |

**Transcript Alterations Specific to the Extensor Digitorum Longus**

| **Accession No.** | **Gene** | **Mean Fold Difference** |
| --- | --- | --- |
| *Transcription* | | |
| NM_053727 | Nuclear factor, interleukin 3 regulated *Nfil3* | 2.3 |
| NM_053536 | Kruppel-like factor 15 *Klf15* | 2.1 |
| NM_012912 | Activating transcription factor 3 *Atf3* | 0.7 |
| -- or  BF415939 | FBJ murine osteosarcoma viral oncogene homolog *Fos* | 0.7 |
| NM_001025137 | Immediate early response 5 *Ier5* | 1.5 |
| NM_001173437 | ChaC, cation transport regulator-like 1 (E. coli) (predicted) *Chac1_predicted* | 1.8 |
| NM_022623 | Frizzled homolog 4 (Drosophila) *Fzd4* | 1.8 |
| NM_001007684 | Kruppel-like factor 2 (lung) (predicted) *Klf2_predicted* | 1.7 |
| NM_133303 /// XM_001074956 /// XM_002729454 | Basic helix-loop-helix domain containing, class B3 *Bhlhb3* | -1.6 |
| NM_080778 | Nuclear receptor subfamily 2, group F, member 2 *Nr2f2* | -1.5 |
| NM_001014035 | Similar to myocyte enhancer factor 2C *Mef2a* | -1.6 |
| NM_001107001 | AF4/FMR2 family, member 4 (predicted) *Aff4* | -2.1 |
| XM_001073964 /// XM_003752366 | Hepatic leukemia factor *Hlf* | -1.5 |
| AI406967 | Early B-cell factor 1 *Ebf1* | -1.6 |
| NM_147136 | rRNA promoter binding protein *LOC257642* | -0.8 |
| AA900904 | Membrane-associated nucleic acid binding protein *Mnab* | -0.8 |
| NM_133615 | TAF9-like RNA polymerase II, TATA box binding protein (TBP)-associated factor, 31kDa *Taf9l* | -0.8 |
| BE099893 | Transcribed locus, moderately similar to XP_580018.1 PREDICTED: hypothetical protein XP_580018 [Rattus norvegicus] *E2F* | -2.0 |
| NM_013060 | Inhibitor of DNA binding 2, *id2* | -1.6 |
| BG670091 | RNA binding motif protein 4 (predicted) *Rbm4* | -1.9 |
| NM_001033701 | Zinc finger homeobox 1b *Zfhx1b* | -1.8 |
| AW435211 | Transcription factor 4 *Tcf4* | -1.9 |
| AW532489 | Zinc finger, DHHC-type containing 21 *Zdhhc21* | -1.8 |
| AI710284 | v-ets erythroblastosis virus E26 oncogene homolog 1 *Ets1* | -2.1 |
| NM_133303 /// XM_001074956 /// XM_002729454 | Basic helix-loop-helix domain containing, class B2 *Bhlhb2* | -1.7 |
| *Stress* | | |
|  |  |  |
| NM_001037365 /// XR_145766 | Brain expressed X-linked 1 *Bex1* | 1.1 |
| NM_031607 | Heat shock 27kD protein family, member 7 (cardiovascular) *Hspb7* | 0.8 |
| NM_181368 | Musculoskeletal, embryonic nuclear protein 1 *Mustn1* | 1.7 |
| AI175728 | Growth arrest-specific 1 *gas1* | -1.6 |
| NM_001130988 | Similar to RIKEN cDNA 0610039G24 gene (predicted) *SLBP* | -1.8 |
| NM_001109292 | Similar to RCK (predicted) *ddx6* | -1.8 |
| *Signal* | | |
| NM_021693 | SNF1-like kinase *Snf1lk* | 1.7 |
| BE108930 | DEAD box protein 24 *DDX24* | 1.9 |
| NM_001107303 | Arrestin domain containing 2 *Arrdc2* | 3.2 |
| AA818910 | Arrestin domain containing 3 *Arrdc3* | 2.1 |
| AA894192 | Tensin *Tns* | 1.8 |
| NM_001024244 | V-set domain containing T cell activation inhibitor 1 *Vtcn1* | 1.0 |
| NM_001044294 | GABA(A) receptor-associated protein like 1 *GABAaapl1* | 1.7 |
| NM_001008365 | Target of myb1 homolog (chicken) *Tom1* | 0.8 |
| AA894192 | Tensin *tns* | 0.8 |
| NM_001012206 | Pleckstrin homology-like domain, family A, member 3 *Phlda3* | 1.7 |
| BM391274 | Lysyl oxidase-like 2 (predicted) *Loxl2* | -1.7 |
| NM_012817 | Insulin-like growth factor binding protein 5 *Igfbp5* | 0.0 |
| BI294974 | Low density lipoprotein receptor *ldlr* | -1.6 |
| NM_001017960 /// NM_031134 | Thyroid hormone receptor alpha *Thra* | -1.6 |
| NM_022267 | Cyclin D2 *Ccnd2* | -1.9 |
| NM_138905 | Phosphatidic acid phosphatase type 2B *Ppap2b* | -0.9 |
| BE329099 | Translocated promoter region *Tpr* | -1.6 |
| NM_133560 | Trafficking protein, kinesin binding 2 *Trak2* | -1.8 |
| NM_001110151 /// NM_152847 | Sorting nexin family member 27 *Snx27* | -2.0 |
| NM_001037492 | Solute carrier family 41, member 3 *Slc41a3* | -1.8 |
| NM_001107658 /// XM_003749226 /// XM_003753540 | Triple functional domain (PTPRF interacting) *Trio* | -1.7 |
| NM_022712 | Transferrin receptor *Tfrc* | -2.0 |
| NM_001017486 | Similar to NipSnap2 protein (Glioblastoma amplified sequence) *NipSnap2* | -1.7 |
| BE108751 | Rho guanine nucleotide exchange factor (GEF) 12 *Arhgef12* | -1.8 |
| NM_001271090 /// XM_002728965 /// XM_003753491 | Myristoylated alanine-rich protein kinase C substrate *MARCKS* | -1.9 |
| XM_002728399 /// XM_003752899 | Similar to palladin *Palld* | -1.9 |
| NM_176075 | Peroxisome proliferative activated receptor, gamma, coactivator 1 beta *Ppargc1b* | -1.9 |
| NM_001037093 | A kinase (PRKA) anchor protein (yotiao) 9 *Akap9* | -1.8 |
| NM_012817 | Insulin-like growth factor binding protein 5 *Igfbp5* | -1.1 |
| NM_001044284 /// XM_003751150 | TSC22 domain family *Tsc22* | -2.2 |
| XM_003750277 /// XM_003754264 | Ataxia, cerebellar, Cayman type (caytaxin) (predicted) *Atcay* | -3.3 |
| *Proteolysis* | | |
| NM_013156 | Cathepsin L *Ctsl* | 2.0 |
| NM_001025140 | Proteasome (prosome, macropain) activator subunit 4 *Psme4* | 1.7 |
| NM_022867 /// XM_002725196 /// XM_002728433 /// XM_003751628 | Microtubule-associated protein 1 light chain 3 beta *Map1lc3b* | 1.7 |
| NM_001100831 | Proteasome (prosome, macropain) 26S subunit, non-ATPase, 8 *Psmd8* | 1.5 |
| NM_001107919 | F-box and leucine-rich repeat protein 4 (predicted) *Fbxl4_predicted* | 1.5 |
| BF416513 | Ubiquitin-conjugating enzyme E2E 2 (UBC4/5 homolog, yeast) *Ube2e2* | -2.0 |
| *Muscle* | | |
| NM_001107589 | Ankyrin repeat domain 2 (stretch responsive muscle) (predicted) *Ankrd2* | 2.9 |
| NM_001191862 | Filamin C, gamma (actin binding protein 280) (predicted) *Flnc_predicted* | 0.7 |
| NM_019217 | Microtubule-associated protein 1b *Map1b* | 1.9 |
| AI105018 | Cardiomyopathy associated 1 (predicted) *Cmya1_predicted* | 1.5 |
| NM_001100964 /// XM_003749707 | Similar to Leiomodin 1 (Leiomodin, muscle form) (64 kDa autoantigen D1) (64 kDa autoantigen 1D) (64 kDa autoantigen 1D3) (Thyroid-associated ophthalmopathy autoantigen) (Smooth muscle leiomodin) (SM-Lmod) *LOC296935* | 1.6 |
| NM_031825 | Fibrillin 1 *Fbn1* | -1.7 |
| NM_001108416 /// XM_003751703 /// XM_003752990 | Supervillin *Svil* | -0.8 |
| *Metabolism* | | |
| NM_138502 | Monoglyceride lipase *Mgll* | -1.3 |
| NM_138898 | Phospholipase B *Phlpb* | 1.7 |
| NM_017073 | Glutamate-ammonia ligase (glutamine synthase) *Glul* | 1.9 |
| NM_001270852 /// NM_001270853 /// NM_012545 | Dopa decarboxylase *Ddc* | 1.9 |
| NM_001014229 | Similar to es 64 *LOC363675* | 1.7 |
| NM_177426 | Glutathione S-transferase, mu 2 *Gstm2* | 1.7 |
| NM_017154 | Xanthine dehydrogenase *Xdh* | 1.9 |
| NM_001135778 | Proline dehydrogenase *Prodh* | 1.8 |
| NM_022525 | Glutathione peroxidase 3 *Gpx3* | 1.8 |
| NM_017073 | Glutamate-ammonia ligase (glutamine synthase) *Glul* | 1.6 |
| AA955605 | Alanine aminotransferase 2 *Alt1* | -1.5 |
| NM_031039 | Glutamic pyruvic transaminase 1, soluble *Gpt1* | -1.6 |
| NM_017206 | Solute carrier family 6 (neurotransmitter transporter, taurine), member 6 *Slc6a6* | -1.7 |
| NM_013128 | Carboxypeptidase E *Cpe* | -1.5 |
| NM_199394 | Ectonucleoside triphosphate diphosphohydrolase 5 *Entpd5* | -1.7 |
| NM_001108937 | PABC1-interacting protein 1 *Paip1* | -1.5 |
| NM_130739 | Acyl-CoA synthetase long-chain family member 6 *Acsl6* | -1.7 |
| AI638990 | Aconitase 2, mitochondrial *Aco2* | -1.8 |
| NM_001106162 | Abhydrolase domain containing 3 (predicted) *Abhd3* | -1.6 |
| NM_022278 | Glutaredoxin 1 (thioltransferase) *Glrx1* | -1.5 |
| AI454322 | Glutamic pyruvate transaminase (alanine aminotransferase) 2 (predicted) *Gpt2* | -1.2 |
| AI070365 | Acyl-CoA synthetase *acyl-CoA synthetase* | -1.8 |
| *Inflammatory* | | |
| NM_019242 | Interferon-related developmental regulator 1 *Ifrd1* | 1.8 |
| NM_053383 | Complement component 1, q subcomponent, receptor 1 *C1qr1* | -1.7 |
| *ECM* | | |
| NM_012649 | Syndecan 4 *Sdc4* | 1.7 |
| AI409738 | Neural cell adhesion molecule 1 *Ncam1* | 1.8 |
| BI289692 | Fraser syndrome 1 *Fras1* | -1.6 |
| *Unclassified* | | |
| NM_134412 /// XR_085760 /// XR_086177 | PMF32 protein *Pmf31* | -1.9 |
| AW524414 | Transcribed locus *lnp* | -1.8 |
| NM_001025042 | Hypothetical protein LOC499856 *LOC499856* | 2.7 |
| XM_001074167 /// XM_574086 | Similar to OTU domain containing 1 (predicted) *RGD1563344_predicted* | 1.9 |
| 1381684_at | Transcribed locus unknown | 1.8 |
| 1373066_at | Transcribed locus unknown | 1.8 |
| 1386669_at | Transcribed locus unknown | 1.9 |
| AI180101 | RGD1565926 (predicted) *RGD1565926_predicted* | 0.8 |
| AI105202 | Transcribed locus, strongly similar to XP_579758.1 PREDICTED: hypothetical protein XP_579758 [Rattus norvegicus] *unknown* | 1.5 |
| 1379707_at | Transcribed locus --- | -1.8 |
| 1384743_at | Transcribed locus --- | -1.6 |
| NM_001108001 | Similar to RIKEN cDNA 2310042D19 *RGD1304931* | -1.8 |
| 1374089_at | Transcribed locus --- | -1.6 |
| 1382472_at | --- | -1.5 |
| 1390436_at | Transcribed locus *unknown* | -0.9 |
| 1385350_at | Transcribed locus *---* | -1.8 |
| XR_146472 /// XR_147198 | --- *Xist* | -1.7 |
| 1395474_at | Transcribed locus --- | -0.9 |
| 1374236_at | Transcribed locus *unknown* | -2.0 |
| --AI030169 | RGD1565240 (predicted) *RGD1565240_predicted* | -1.7 |
| NM_001108180 | Transcribed locus *ky* | -2.0 |
| XR_145787 /// XR_145788 /// XR_146772 /// XR_146773 | CDNA clone IMAGE:7321089 *Hepcarcin* | -1.9 |
| AW535897 | Transcribed locus *AUTS2* | -2.0 |
| NM_001191826 /// XM_003751050 | Transcribed locus *Gramd1c* | 0.4 |
| 1392962_at | --- *unknown* | -2.1 |
| BG666712 | --- *m6b* | -1.1 |

**Transcript Alterations Shared Between Extensor Digitorum Logus and the Diaphragm**

| **Accession No.** | **Gene** | **Mean Fold Difference** |
| --- | --- | --- |
| *Transcription* | | |
| BF406350 | Forkhead box O1A *Foxo1a* | 2.6 |
| NM_133290 | Zinc finger protein 36 *Zfp36* | 2.4 |
| NM_053857 | Eukaryotic translation initiation factor 4E binding protein 1  *Eif4ebp1* | 2.1 |
| NM_001105880 | Zinc finger and BTB domain containing 20 *Zbtb20* | -2.0 |
| -- or BF396723 | DNA (cytosine-5-)-methyltransferase 3 alpha *Dnmt3a* | -2.0 |
| *Stress* | | |
| NM_080782 | Cyclin-dependent kinase inhibitor 1A *Cdkn1a* | 3.3 |
| NM_001008321 | Growth arrest and DNA-damage-inducible 45 beta *Gadd45b* | 1.8 |
| NM_001106396 | Sestrin 1 (predicted) *Sesn1_predicted* | 2.0 |
| NM_001008321 | Growth arrest and DNA-damage-inducible 45 gamma *Gadd45g* | 1.9 |
| *Signal* | | |
| NM_001007738 | Similar to hypothetical protein MGC34760 *RGD1359349* | 2.2 |
| NM_022602 | Serine/threonine-protein kinase pim-3 *Pim3* | 2.0 |
| NM_001106838 | Similar to hypothetical protein MGC6835 *DEPP-like* | 2.1 |
| -- or BE329364 | Solute carrier family 7 *Slc7a2* | 2.0 |
| NM_022712 | Transferrin receptor *Tfrc* | -1.9 |
| -- or AA924336 | Mitochondrial tumor suppressor 1 *Mtus1* | -2.2 |
| -- or AI102833 | Cyclin-dependent kinase 6 *cdk6* | -2.2 |
| NM_031612 | Apelin, AGTRL1 ligand *Apln* | -2.3 |
| NM_178096 | Neuronal regeneration related protein *Nrep* | -2.9 |
| -- or  AI071994 | Dickkopf homolog 4 *Dkk4* | -2.6 |
| *Proteolysis* | | |
| NM_080903 /// XM_003750058 | Tripartite motif protein 63 *Trim63* | 2.1 |
| -- or AA925542 | F-box protein 32 *FBXO32* | 2.3 |
| *Muscle* | | |
| NM_057144 | Cysteine and glycine-rich protein 3 *Csrp3* | 2.4 |
| NM_001108180 | Kyphoscoliosis peptidase *Ky* | -2.2 |
| *Metabolism* | | |
| NM_144738 /// XM_003749429 | Neuropathy target esterase like 1 *Ntel1* | 2.6 |
| NM_001047913 | CDNA clone IMAGE:7455024 *SQRDL* | 2.0 |
| NM_031544 | Adenosine monophosphate deaminase 3 *Ampd3* | 2.4 |
| NM_053477 | Malonyl-CoA decarboxylase *Mlycd* | 2.0 |
| NM_031834 | Sulfotransferase family 1A, phenol-preferring, member 1 *Sult1a1* | 2.0 |
| XM_001075627 /// XM_003748835 | Cytochrome c oxidase, subunit VIIa 1 *CoxVII* | -1.9 |
| NM_001207007 /// NM_012793 | Guanidinoacetate methyltransferase *Gamt* | -2.0 |
| *ECM* | | |
| NM_031050 | Lumican *Lum* | -1.8 |
| NM_032085 | Procollagen, type III, alpha 1 *Col3a1* | -2.0 |
| NM_053356 /// XM_003749690 /// XM_003749691 | Procollagen, type I, alpha 2 *Col1a2* | -1.9 |
| NM_001100535 /// XM_001066530 /// XM_003749933 /// XM_003749934 /// XM_003754014 /// XM_216399 | Procollagen, type XV *Col15a1* | -2.0 |
| NM_053304 | Procollagen, type 1, alpha 1 *Col1a1* | -2.4 |
| *Unclassified* | | |
| XR_146198 /// XR_146518 | RGD1565595 (predicted) *RGD1565595_predicted* | 3.8 |
| NM_001025042 | Hypothetical protein LOC499856 *LOC499856* | 2.4 |
| XM_003750594 | RGD1561460 (predicted) *RGD1561460_predicted* | 2.0 |
| AA818910 | Transcribed locus *Arrdc3* | 2.6 |
| XR_146396 | Uncharacterized | 2.1 |
| NM_001025129 | Similar to downregulated in renal cell carcinoma *RGD1306327* | 1.9 |
| BF386502 | Non coding transcript | 1.2 |
| BF392753 | Clone UI-R-CA0-baw-a-05-0-U | -1.8 |
| BF420311 | Transcribed locus *AUTS2* | -2.4 |
| XR_360313 | Uncharacterized transcript | -2.3 |

**Transcript Alterations Specific to Extraocular Muscle**

| **Accession No.** | **Gene** | **Mean Fold Difference** |
| --- | --- | --- |
| *Transcription* | | |
| NM_017325 | Runt related transcription factor 1 *Runx1* | 2.2 |
| NM_013154 | CCAAT/enhancer binding protein (C/EBP), beta *Cebpb* | 2.0 |
| BG371725 | Zinc finger and BTB domain containing 16 *Zbtb16* | 1.9 |
| NM_053449 /// XM_001067733 /// XM_343629 | Histone deacetylase 4 (predicted) *Hdac4* | 0.7 |
| *Signal* | | |
| AI145227 | Similar to male sterility domain containing 1 (predicted) *sterility domain containing 1* | 4.1 |
| NM_012703 | Thyroid hormone responsive protein *Thrsp* | 4.0 |
| NM_001106058 | Claudin 10 (predicted) *Cldn10* | 2.5 |
| NM_001108693 | Similar to retinoid binding protein 7 (predicted) *Rbp7* | 1.9 |
| NM_001270954 /// XM_001077321 /// XM_340809 | RAS, dexamethasone-induced 1 *Rasd1* | 1.8 |
| NM_001270807 /// NM_001270808 /// NM_001270809 /// NM_001270810 /// NM_001270811 /// NM_133386 | Sphingosine kinase 1 *Sphk1* | 2.5 |
| NM_001107335 | Death associated protein kinase 1 (predicted) *Dapk1* | 3.5 |
| NM_001107602 | Elongation of very long chain fatty acids (FEN1/Elo2, SUR4/Elo3, yeast)-like 3 (predicted) *Elovl3_predicted* | 1.5 |
| NM_001025152 | Membrane targeting (tandem) C2 domain containing 1 *Mtac2d1* | 2.0 |
| NM_001007144 | Adipose differentiation related protein *Adfp* | 1.7 |
| NM_145878 | Fatty acid binding protein 5, epidermal *Fabp5* | 0.1 |
| NM_139095 | Transmembrane protein 37 *Tmem37* | 2.5 |
| BE120535 | Stomatin (Epb7.2)-like 3 (predicted) *Stoml3_predicted* | 0.9 |
| NM_001108497 | Similar to C11orf17 protein (predicted) *RGD1306959_predicted* | 0.8 |
| NM_001108497 | Similar to C11orf17 protein (predicted) *bca3* | 0.8 |
| NM_001034152 | HMP19 protein *MGC125201* | 0.8 |
| NM_207602 | ST3 beta-galactoside alpha-2,3-sialyltransferase 6 *St3gal6* | -1.6 |
| NM_053750 | Natriuretic peptide precursor type C *Nppc* | -1.9 |
| NM_133527 | Folate receptor 1 (adult) *Folr1* | -0.9 |
| NM_001107464 | Dapper homolog 2, antagonist of beta-catenin (xenopus) (predicted) *Dact2_predicted* | -2.4 |
| NM_001009639 | Similar to RIKEN cDNA 2700055K07 *RGD1305061* | -2.5 |
| XM_001071670 /// XM_213610 | Protein tyrosine phosphatase-like (proline instead of catalytic arginine), member b (predicted) *Ptplb* | 1.0 |
| *Proteolysis* | | |
| NM_012938 | Cathepsin E *Ctse* | 2.8 |
| NM_012593 | Kallikrein 7 *Klk7* | 2.6 |
| NM_001005549 | Tubulointerstitial nephritis antigen *Tinag* | -1.1 |
| NM_001108769 | Similar to F-box protein FBL2 (predicted) *RGD1311830_predicted* | -3.4 |
| *Muscle* | | |
| NM_024485 | Cholinergic receptor, nicotinic, alpha polypeptide 1 (muscle) *Chrna1* | 2.0 |
| NM_206851 | SET and MYND domain containing 2 *Smyd2* | 1.6 |
| NM_057208 /// NM_173111 | Tropomyosin 3, gamma *Tpm3* | -1.6 |
| *Metabolism* | | |
| NM_020538 | Arylacetamide deacetylase (esterase) *Aadac* | 5.1 |
| NM_001108803 | Monoacylglycerol O-acyltransferase 1 (predicted) *Mogat1_predicted* | 3.2 |
| NM_019286 | Alcohol dehydrogenase 1 (class I) *Adh1* | 3.1 |
| NM_134389 | Acyl-CoA synthetase bubblegum family member 1 *Acsbg1* | 2.0 |
| NM_134349 | Microsomal glutathione S-transferase 1 *Mgst1* | 2.1 |
| NM_133295 | Carboxylesterase 3 *Ces3* | 1.0 |
| NM_053433 | Flavin containing monooxygenase 3 *Fmo3* | 2.4 |
| NM_013015 | Prostaglandin D2 synthase *Ptgds* | -0.5 |
| NM_001012345 | Diacylglycerol O-acyltransferase homolog 2 (mouse) *Dgat2* | 2.0 |
| NM_017332 | Fatty acid synthase *Fasn* | -0.6 |
| NM_133606 | Enoyl-Coenzyme A, hydratase/3-hydroxyacyl Coenzyme A dehydrogenase *Ehhadh* | 1.8 |
| NM_053607 | Acyl-CoA synthetase long-chain family member 5 *Acsl5* | -0.1 |
| NM_001108406 | Serine palmitoyltransferase, long chain base subunit 1 (predicted) *Sptlc1_predicted* | 1.5 |
| BI288055 | Transcribed locus, moderately similar to NP_598623.1 fibrinogen, gamma polypeptide [Mus musculus] *Bdh1* | -0.5 |
| *Inflammatory* | | |
| NM_012881 | Secreted phosphoprotein 1 *Spp1* | 3.0 |
| NM_016994 | Complement component 3 *C3* | 1.0 |
| NM_053587 | S100 calcium binding protein A9 (calgranulin B) *S100a9* | 1.8 |
| NM_031530 | Chemokine (C-C motif) ligand 2 *Ccl2* | -1.7 |
| BG378317 | Tnfrsf19 *Tnfrsf19* | -1.8 |
| NM_001033882 /// NM_001033883 /// NM_022177 | Chemokine (C-X-C motif) ligand 12 *Cxcl12* | -1.8 |
| *ECM* | | |
| AI070875 | Matrix Gla protein *Mgp* | 1.9 |
| NM_001100841 | Laminin, beta 3 *Lamb3* | -1.7 |
| *Unclassified* | | |
| NM_001193275 | Similar to epididymal protein (predicted) *RGD1561290_predicted* | 2.7 |
| XM_006222052 | dopachrome tautomerase *Dct* | 1.6 |
| NM_001107561 | Secretoglobin, family 1C, member 1 (predicted) *Scgb1c1* | 1.6 |
| BG381207 | Non-coding | 2.2 |
| XM_006224256 | PREDICTED: Rattus norvegicus collagen, type XXV, alpha 1 (Col25a1), transcript variant X1, mRNA | 2.2 |
| BF553180 | CLONE=UI-R-C2-nj-e-01-0-UI | 2.1 |
| NM_001135016 | Transcribed locus | 1.8 |
| AI716904 | RGD1563308 (predicted) *RGD1563308_predicted* | 1.7 |
| BM388725 | Transcribed locus --- | 1.9 |
| BF565662 | Transcribed locus --- | 2.2 |
| BF386199 | Transcribed locus --- | 1.9 |
| AW526307 | Transcribed locus --- | 1.6 |
| NP_954525 | Predicted tubulin gene product | 1.5 |
| NM_001025008 | Hypothetical protein LOC314996 *un* | 0.2 |
| NM_001108100 | Tyrosine phosphorylation regulated kinase *Dyrk2* | -1.9 |
| XM_002726617 /// XM_002729572 | Similar to hypothetical protein FLJ37118 (predicted) *RGD1560874_predicted* | -1.9 |

**Transcript Alterations Shared Between Extraocular Muscle and Diaphragm**

| **Accession No.** | **Gene** | **Mean Fold Difference** |
| --- | --- | --- |
| *Stress* | | |
| NM_031971 | Heat shock 70kD protein 1B (mapped) *Hspa1b* | -1.7 |
| NM_031971 /// NM_212504 | Heat shock 70kD protein 1A /// heat shock 70kD protein 1B (mapped) *Hspa1a* | -2.0 |
| *Signal* | | |
| NM_012588 | Insulin-like growth factor binding protein 3 *Igfbp3* | 2.7 |
| NM_001008217 | LSM14A, SCD6 homolog *LSM14* | -2.2 |
| NM_013197 | Aminolevulinic acid synthase 2 *Alas2* | -2.7 |
| NM_053338 | Ras-related associated with diabetes *Rrad* | -2.3 |
| *Muscle* | | |
| NM_177425 | Cysteine and glycine-rich protein 2 *Csrp2* | 2.1 |
| *Metabolism* | | |
| NM_019363 | Aldehyde oxidase 1 *Aox1* | 2.5 |
| BF283381 | 6-phosphofructo-2-kinase/fructose-2,6-biphosphatase 3 *Pfkfb3* | 2.5 |
| NM_016998 | Carboxypeptidase A1 *Cpa1* | -2.0 |
| NM_001034124 /// XM_003750842 | Microfibrillar-associated protein 4 *Mfap4* | -2.1 |

**Transcript Alterations Shared Between Extraocular Muscle and Extensor Digitorum Longus**

| **Accession No.** | **Gene** | **Mean Fold Difference** |
| --- | --- | --- |
| *Signal* | | |
| AI101194 | Spermatid perinuclear RNA binding protein *Spnr* | -2.0 |
| BE115141 | Phosphoinositide-3-kinase, regulatory subunit 1 *Pik3r1* | 2.1 |
| BM387773 | Wingless-related MMTV integration site 16 *Wnt16* | -2.2 |
| *Muscle* | | |
| NM_019217 | Microtubule-associated protein 1b *Map1b* | 2.0 |
| *Metabolic* | | |
| NM_013167 | Uncoupling protein 3 (mitochondrial, proton carrier) *Ucp3* | 3.1 |
| NM_001270961 /// NM_012532 | Ceruloplasmin *Cp* | 1.9 |
| *Inflammatory* | | |
| NM_181086 | Tumor necrosis factor receptor superfamily, member 12a *Tnfrsf12a* | 2.2 |
| *Unclassified* | | |
| AA942745 | Transcribed locus *EST* | 2.0 |

**Transcript Alterations Shared Among All Muscles**

| **Accession No.** | **Gene** | **Mean Fold Difference** |
| --- | --- | --- |
| *Transcription regulation* | | |
| NM_013220 | Ankyrin repeat domain 1 (cardiac muscle) *Ankrd1* | 4.9 |
| NM_024125 | CCAAT/enhancer binding protein (C/EBP), delta *Cebpd* | 4.6 |
| AI555855 | Nuclear factor I/X (CCAAT-binding transcription factor) *nfix* | -3.2 |
| NM_001105720 | Nuclear factor of kappa light chain gene enhancer in B-cells inhibitor, alpha *Nfkbia* | 2.5 |
| *Stress* | | |
| NM_080906 | DNA-damage-inducible transcript 4 *Ddit4* | 3.6 |
| NM_024127 | Growth arrest and DNA-damage-inducible 45 alpha *Gadd45a* | 2.3 |
| NM_012681 /// NM_138826 /// XM_002730233 /// XM_003754778 | Metallothionein 1a *Mt1a* | 5.4 |
| NM_001014071 | ERBB receptor feedback inhibitor 1 *Errfi1* | 2.9 |
| NM_001137564 | Metallothionein 1e *Mt1e* | 3.8 |
| *Signal* | | |
| NM_133298 | Glycoprotein (transmembrane) nmb *Gpnmb* | 3.5 |
| BF550565 | Golgi- associated PR-1 protein *Glipr2* | -2.3 |
| NM_001108100 | Dual specificity tyrosine-phosphorylation-regulated kinase 2 *Dyrk2* | -2.4 |
| NM_001003401 | Ectodermal-neural cortex 1 *Enc1* | 2.2 |
| *Metabolism* | | |
| NM_001111269 /// NM_001113223 | Hemoglobin beta chain complex *Hbb* | -3.7 |
| NM_017130 | Neuraminidase 2 *Neu2* | -3.0 |
| NM_001007722 /// NM_013096 | Hemoglobin alpha, adult chain 1 /// hemoglobin alpha 2 chain *Hba-a1* | -3.1 |
| NM_001108509 /// XM_003749014 /// XM_003749015 | Patatin-like phospholipase domain containing 2 (predicted) *Pnpla2* | 2.5 |
| NM_053551 | Pyruvate dehydrogenase *pdk4* | 2.7 |
| -- or AA859079 | Flavin containing monooxygenase 2 *fmo2* | 3.6 |
| -- or BI276554 | GalNAc transferase 12 *galnt12* | 2.9 |
| *Immune response* | | |
| NM_001012174 | FK506 binding protein 5 *Fkbp5* | 5.1 |
| *ECM* | | |
| NM_199115 | Angiopoietin-like 4 *Angptl4* | 10.1 |
